# A chromosome-level reference genome assembly for Gilbert’s skink *Plestiodon gilberti*

**DOI:** 10.1093/jhered/esaf040

**Published:** 2025-06-23

**Authors:** Jonathan Q Richmond, Merly Escalona, Mohan P A Marimuthu, Oanh Nguyen, Samuel Sacco, Eric Beraut, Erin Toffelmier, Benjamin R Karin, Robert D Cooper, Robert N Fisher, Ian J Wang, H Bradley Shaffer

**Affiliations:** U.S. Geological Survey, Western Ecological Research Center, 4165 Spruance Rd. Suite 200, San Diego, CA, United States; Department of Biomolecular Engineering, University of California, Santa Cruz, CA, United States; DNA Technologies and Expression Analysis Core Laboratory, Genome Center, University of California, Davis, CA, United States; DNA Technologies and Expression Analysis Core Laboratory, Genome Center, University of California, Davis, CA, United States; Department of Ecology and Evolutionary Biology, University of California, Santa Cruz, CA, United States; Department of Ecology and Evolutionary Biology, University of California, Santa Cruz, CA, United States; Department of Ecology & Evolutionary Biology, University of California, Los Angeles, CA, United States; La Kretz Center for California Conservation Science, Institute of the Environment and Sustainability, University of California, Los Angeles, CA, United States; Department of Environmental Science, Policy, and Management, University of California, Berkeley, CA, United States; Department of Ecology & Evolutionary Biology, University of California, Los Angeles, CA, United States; La Kretz Center for California Conservation Science, Institute of the Environment and Sustainability, University of California, Los Angeles, CA, United States; U.S. Geological Survey, Western Ecological Research Center, 4165 Spruance Rd. Suite 200, San Diego, CA, United States; Department of Environmental Science, Policy, and Management, University of California, Berkeley, CA, United States; Museum of Vertebrate Zoology, University of California, Berkeley, CA, United States; Department of Ecology & Evolutionary Biology, University of California, Los Angeles, CA, United States; La Kretz Center for California Conservation Science, Institute of the Environment and Sustainability, University of California, Los Angeles, CA, United States

**Keywords:** adaptive introgression, California Conservation Genomics Project, conservation genetics, ecological speciation, genome assembly, Scincidae

## Abstract

Advances in genomic studies are revealing that gene flow between species is more frequent than previously understood, although the ways in which hybridization can bias gene flow across species boundaries or the extent to which introgression might be adaptive remain unexplored in most systems. We report on an annotated chromosome-level genome assembly for the Gilbert’s skink, *Plestiodon gilberti*, one of 18 clades of reptiles and amphibians selected for reference genome sequencing in the California Conservation Genomics Project. This assembly was produced using Pacific Biosciences HiFi long reads and Omni-C proximity ligation data. Although members of the Scincidae comprise nearly one-quarter of all lizard species (1785 described species), this de novo assembly represents one of only 10 skink species globally and the first North American skink with a reference genome. The assembly has a total length of ~ 1.57 Gb, a scaffold N50 length of ~ 231.32 Mb, read coverage of ~56X, and BUSCO completeness score of 97.2% based on the Tetrapoda ortholog database. *Plestiodon gilberti* is a member of the *Plestiodon skiltonianus* species complex, a group with many of the characteristics of ecological speciation but where ancient hybridization and biased introgression present challenges to retracing the initial patterns of lineage divergence. Combined with dense sampling of resequenced genomes in the California Conservation Genomics Project, including other members of the *P. skiltonianus* complex, this reference genome will enable future analyses of the links between divergent selection and the genes underlying speciation, as well as the potential for introgression to enable adaptation to new or changing environments.

The California Floristic Province (CFP) is a region of high endemism and high species-level diversity, in part because of its wide variety of contained ecosystems, variable climate conditions, and topographic heterogeneity (Myers et al. 2000; Mittermeier et al. 2004; Brooks et al. 2006; Baldwin 2014). These and other factors have influenced population- and species-level diversification in a variety of plant and animal taxa, with numerous comparative studies identifying hotspots for genetic diversity and differentiation within the CFP (Beninde et al. 2022b; Calsbeek et al. 2003; Chatzimanolis and Caterino 2007; Feldman and Spicer 2006; Rissler et al. 2006; Vandergast et al. 2008). For many of these taxa, the extent to which neutral processes or natural selection influenced diversification remains poorly understood, although considerable evidence now points to natural selection as a general, leading mechanism of speciation in plants and animals (Coyne and Orr 2004; Schluter 2009; Nosil 2012). Despite this understanding, much remains to be discovered about the specific genes and genetic mechanisms that underlie adaptive trait divergence and how these traits influence gene flow between ecologically divergent populations (Pavey et al. 2010; Wolf et al. 2010; Schluter and Rieseberg 2022).

Scincid lizards (family Scincidae) of the *Plestiodon skiltonianus* species complex show many characteristics consistent with ecological speciation (Coyne and Orr 2004; Schluter 2009), including the presence of a size-assortative pre-mating barrier that genetically isolates species occupying different thermal niches (Richmond et al. 2011; Wogan and Richmond 2015). As with many speciation studies, ‘leaky’ reproductive barriers can complicate the ability to retrace the history of divergence between closely related taxa—in this case, a recent genomic study uncovered evidence of separate, ancient hybridization events, the effects of which led to extreme mitonuclear discordance and biased gene flow from a single small-bodied species (*P. skiltonianus*) into separate lineages of a large-bodied taxon (*P. gilberti*) within the complex (Richmond et al. 2025). This raises questions relating to the extent and context of introgression, specifically whether introgressed variation is localized to certain parts of the genome and whether it is adaptive in recipient species.

Here, we report on the first reference genome for *Plestiodon gilberti* (Fig. 1). This is the first genome for a North American skink (https://www.ncbi.nlm.nih.gov/datasets/genome; accessed on 4 December 2024). It is also one of over 150 species projects included in the California Conservation Genomics Project (CCGP), a state-funded program generating a comprehensive genomic database to help manage California’s biodiversity in the face of climate change and other anthropogenic challenges (Beninde et al. 2022a; Fiedler et al. 2022; Shaffer et al. 2022; Toffelmier et al. 2022). This new, annotated genome, combined with dense sampling of resequenced *Plestiodon* genomes across California that is also part of the CCGP, will provide new opportunities to address questions about the genes underlying adaptive trait divergence, including the role of introgressed polymorphisms in speciation and local adaptation. Because of the possible links between thermal adaptation and speciation in this group, answering questions about speciation genes may also be relevant to determining whether standing genetic variation might provide a means for rapid response to a changing climate.

**Figure 1:**
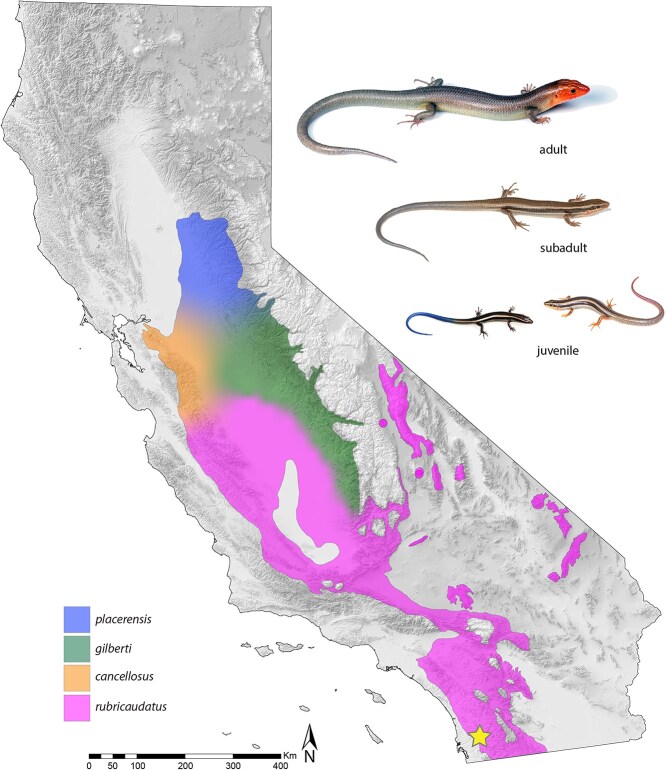
Distribution of the Gilbert’s skink, *Plestiodon gilberti*, with colors representing each of four currently recognized subspecies in California. The yellow star indicates the collection site for the voucher specimen in Deer Park, California (San Diego County). Images show the ontogeny of the color pattern: Uniform-colored adult male with reddish-orange breeding colors (top; photo by J. Shedd), a subadult with faded lateral striping (middle; photo by J. Shedd), and juveniles showing two variations of decoy coloration on the tail (blue; photo by R. Hansen: Pink; photo by J. Richmond). Map base layer obtained from a Shuttle Radar Topography Mission digital elevation model provided by the U.S. Geological Survey’s Earth Resources Observation and Science (EROS) Center (DOI: 10.5066/F7PR7TFT).

## Methods

### Biological materials

There are currently four recognized subspecies of *P. gilberti* in California (Hansen and Shedd 2025: Fig. 1). We captured an adult female *P. gilberti rubricaudatus* in Deer Park in San Diego County, California (Fig. 1: 33.2444°N, -117.1449°W) on 8 September 2020 (CDFW permit# SC-838 USGS Entity Permit). After euthanization, we flash froze multiple tissue samples (blood, brain, intestine liver, lung, muscle, and ovary) in liquid nitrogen, stored them at −80°C, and provided subsamples to the University of California (UC) Davis DNA Technologies and Expression Analysis Core Facility, the Paleogenomics Lab at UC Santa Cruz, and the UC Los Angeles Technology Center for Genomics & Bioinformatics for DNA/RNA extractions (refer to descriptions below for the roles of each facility in completing the project). The voucher specimen was preserved in 10% buffered formalin and is accessioned at the Museum of Vertebrate Zoology (MVZ:Herp:300830).

### High-molecular-weight genomic DNA isolation

High molecular weight genomic DNA was extracted from 26 mg of liver tissue using the Nanobind Tissue Big DNA kit following the manufacturer's protocol (Pacific BioSciences—PacBio, Menlo Park, CA). We estimated DNA purity (i.e. 260/280 = 1.83 and 260/230 = 2.24) using a NanoDrop ND-1000 spectrophotometer and quantified the final DNA yield (34 μg) using the Quantus Fluorometer (QuantiFluor ONE dsDNA Dye assay; Promega, Madison, WI). We estimated the fragment size distribution of the extracted genomic DNA using the Femto Pulse system (Agilent, Santa Clara, CA) and found that 58% the fragments were 100 Kb or longer.

### HiFi library preparation and sequencing

We constructed a HiFi SMRTbell library at the UC Davis DNA Technologies and Expression Analysis Core Facility using the SMRTbell Express Template Prep Kit v2.0 (PacBio, Menlo Park, CA; Cat. #100-938-900) following the manufacturer's instructions. We began by shearing high molecular weight gDNA to a target size of 15–20 kb using Diagenode’s Megaruptor 3 system (Diagenode, Belgium; Cat. #B06010003). We then concentrated the sheared DNA using 0.45X AMPure PB beads (PacBio, Menlo Park, CA; Cat. #100-265-900) for removal of single-strand overhangs at 37°C for 15 min. Next, we performed enzymatic steps of DNA damage repair at 37°C for 30 min, end repair and A-tailing at 20°C for 10 min then at 65°C for 30 min, ligation of overhang adapters v3 at 20°C for 60 min, and then inactivated the ligase at 65°C for 10 min. We purified the library using 0.45X AMPure PB beads, and size-selected fragments in the 7–9 kb range using a BluePippin/PippinHT system (Sage Science, Beverly, MA; Cat #BLF7510/HPE7510). Finally, we sequenced the HiFi SMRTbell library at the same core facility at UC Davis using four 8 M SMRT cells (PacBio, Menlo Park, CA; Cat #101-389-001) on the PacBio Sequel IIe with sequencing chemistry 2.0 and 30-h movies.

### Omni-C library preparation

We prepared the Omni-C library at the UC Santa Cruz Paleogenomics Lab using the Dovetail Omni-C Kit (Dovetail Genomics, CA) according to the manufacturer’s protocol with slight modifications. First, we ground specimen tissue with a mortar and pestle on liquid nitrogen and then fixed chromatin in place in the nucleus. The suspended chromatin solution was then passed through 100 and 40 μm cell strainers to remove large debris. We digested fixed chromatin under various conditions of DNase I until we obtained a suitable fragment length distribution of DNA molecules. We repaired and ligated chromatin ends to a biotinylated bridge adapter followed by proximity ligation of adapter containing ends. After proximity ligation, we reversed crosslinks and purified the DNA by removing proteins and biotin that was not internal to ligated fragments. We generated an NGS library using an NEB Ultra II DNA Library Prep kit (New England Biolabs, Ipswich, MA) with an Illumina compatible y-adaptor. Biotin-containing fragments were then captured using streptavidin beads. We split the post-capture product into two replicates prior to PCR enrichment to preserve library complexity with each replicate receiving unique dual indices. We sequenced the library on an Illumina NovaSeq S4 (Illumina, San Diego, CA) at the Vincent J. Coates Genomics Sequencing Laboratory at UC Berkeley and generated approximately 100 million 2 x 150 bp read pairs per GB of genome length.

### Transcriptome library preparation and sequencing

We extracted total RNA from seven tissues (brain, heart, liver, lung, blood, ovary, and muscle) using a Qiagen RNeasy Mini Kit (Qiagen, Netherlands) according to the manufacturer’s protocol. We then prepared RNA libraries using the KAPA mRNA HyperPrep Kit (Roche, Switzerland) according to the manufacturer’s protocol. Libraries were prepared and sequenced at the UCLA Technology Center for Genomics & Bioinformatics using 150 bp paired end reads on an Illumina NovaSeq X platform (Illumina, San Diego, CA) in a 25B flowcell to generate approximately 50 M reads per library.

### Nuclear genome assembly

We assembled the genome of *P. gilberti* following the CCGP assembly pipeline Version 5.0, as outlined in Table 1, which lists the tools and non-default parameters used in the assembly process. The pipeline uses PacBio HiFi reads and Omni-C data to produce high quality and highly contiguous genome assemblies. First, we removed remnants adapter sequences from the PacBio HiFi dataset using HiFiAdapterFilt (Sim et al. 2022) and generated the initial dual or partially phased diploid assembly (http://lh3.github.io/2021/10/10/introducing-dual-assembly) using HiFiasm (Cheng et al. 2022) in Hi-C mode with the filtered PacBio HiFi reads and the Omni-C dataset. This generates two assemblies, one per haplotype. We then aligned the Omni-C data to both assemblies separately following the Arima Genomics Mapping Pipeline (https://github.com/ArimaGenomics/mapping_pipeline) and scaffolded both assemblies using SALSA (Ghurye et al. 2017; Ghurye et al. 2019).

**Table 1:** Assembly pipeline and software used to generate the *Plestiodon gilberti* reference genome. Software citations are provided in the text.

**Assembly step**	**Software and any non-default options**	**Version**	**Reference**
**Initial assembly**			
Filtering PacBio HiFi adapters	HiFiAdapterFilt	Commit 64d1c7b	Sim et al. 2022
K-mer counting	Meryl (*k* = 21)	1	https://github.com/marbl/meryl
Estimation of genome size and heterozygosity	GenomeScope	2	Ranallo-Benavidez et al. 2020
De novo *assembly (contiging)*	HiFiasm (Hi-C Mode, −primary, output hic.hap1.p_ctg, hic.hap2.p_ctg)	0.16.1-r375	Cheng et al. 2022
**Scaffolding**			
Omni-C data alignment	*Arima Genomics Mapping Pipeline*	Commit 2e74ea4	https://github.com/ArimaGenomics/mapping_pipeline
*Arima Genomics Mapping Pipeline (AGMP)*	BWA-MEM	0.7.17-r1188	Li 2013
	samtools	1.11	Danecek et al. 2011
	filter_five_end.pl (AGMP)	Commit 2e74ea4	https://github.com/ArimaGenomics/mapping_pipeline
	two_read_bam_combiner.pl (AGMP)	Commit 2e74ea4	https://github.com/ArimaGenomics/mapping_pipeline
	picard	2.27.5	https://broadinstitute.github.io/picard/
Omni-C Scaffolding	SALSA (-DNASE, −i 20, −p yes)	2	Ghurye et al. 2017, Ghurye et al. 2019
**Omni-C Contact map generation**			
Short-read alignment	BWA-MEM (-5SP)	0.7.17-r1188	Li 2013
SAM/BAM processing	samtools	1.11	Danecek et al. 2011
SAM/BAM filtering	pairtools	0.3.0	Open2C et al. 2024
Pairs indexing	pairix	0.3.7	Lee et al. 2022
Matrix generation	cooler	0.8.10	Abdennur and Mirny 2019
Matrix balancing	hicExplorer (hicCorrectmatrix correct --filterThreshold −2 4)	3.6	Ramírez et al. 2018
Contact map visualization	HiGlass	2.1.11	Kerpedjiev et al. 2018
	PretextMap	0.1.4	https://github.com/wtsi-hpag/PretextView
	PretextView	0.1.5	https://github.com/wtsi-hpag/PretextMap
	PretextSnapshot	0.0.3	https://github.com/wtsi-hpag/PretextSnapshot
Manual curation tools	Rapid curation pipeline (Wellcome Trust Sanger Institute, Genome Reference Informatics Team)	Commit 7acf220c	https://gitlab.com/wtsi-grit/rapid-curation
**Genome quality assessment**			
Basic assembly metrics	QUAST (−-est-ref-size)	5.0.2	Gurevich et al. 2013
Assembly completeness	BUSCO (−m geno, −l tetrapoda)	5.0.0	Manni et al. 2021
	Merqury	29 January 2020	Rhie et al. 2020
**Contamination screening**			
Local alignment tool	BLAST+ (−db nt, −outfmt '6 qseqid staxids bitscore std', −max_target_seqs 1, −max_hsps 1, −evalue 1e-25)	2.15	Camacho et al. 2009
General contamination screening	BlobToolKit (HiFi coverage, BUSCO = tetrapoda, NCBI Taxa ID = 463,524)	2.3.3	Challis et al. 2020
**Mitochondrial assembly**			
Mitochondrial genome assembly	MitoHiFi (−r, −p 90, −o 1) Reference: Plestiodon capito (NCBI:MK370740.1)	2.2	Uliano-Silva et al. 2023
de novo assembly of mitochondrial long reads	HiFiasm (output bp.p_ctg)	0.24.0-r703	Cheng et al. 2022
Mitochondrial genome annotation	MitoFinder (−o 1, −p 90, MK370740.1.gb)	1.4	Allio et al. 2020

We manually curated both genome assemblies by iteratively generating and analyzing their corresponding Omni-C contact maps. To generate the contact maps we aligned the Omni-C data with BWA-MEM (Li 2013), identified ligation junctions, and generated Omni-C pairs using pairtools (Goloborodko et al. 2018). We then generated a multi-resolution Omni-C matrix with cooler (Abdennur and Mirny 2019) and balanced it with hicExplorer (Ramírez et al. 2018), after which we used HiGlass (Kerpedjiev et al. 2018) and PretextSuite (https://github.com/wtsi-hpag/PretextView; https://github.com/wtsi-hpag/PretextMap; https://github.com/wtsi-hpag/PretextSnapshot) to visualize the contact maps for identifying misassemblies and misjoins. Last, we modified the assemblies using the Rapid Curation pipeline from the Wellcome Sanger Institute, Genome Reference Informatics Team (https://gitlab.com/wtsi-grit/rapid-curation). Remaining gaps (joins generated during scaffolding and curation) were closed using the PacBio HiFi reads and YAGCloser (https://github.com/merlyescalona/yagcloser). Finally, we checked for contamination using the BlobToolKit Framework (Challis et al. 2020).

### Genome quality assembly assessment

We generated k-mer counts from the PacBio HiFi reads using meryl (https://github.com/marbl/meryl). The k-mer counts were then used in GenomeScope 2.0 (Ranallo-Benavidez et al. 2020) to estimate genome features including size, heterozygosity, and repeat content. To obtain general contiguity metrics, we ran QUAST (Gurevich et al. 2013). To evaluate genome quality and functional completeness we used BUSCO (Manni et al. 2021) with the Tetrapoda ortholog database (tetrapoda_odb10) which contains 5286 genes. We performed an assessment of base level accuracy (QV) and k-mer completeness using the previously generated meryl database and merqury (Rhie et al. 2020), and estimated genome assembly accuracy via BUSCO gene set frameshift analysis using the pipeline described in Korlach et al. (2017). Size estimates of the phased blocks were based on contig sizes generated by HiFiasm on HiC mode. We follow the nomenclature for quality metrics established by Rhie et al. (2021), with the genome quality code x.y.P.Q.C, where, x = log10[contig NG50]; y = log10[scaffold NG50]; P = log10 [phased block NG50]; Q = Phred base accuracy QV (quality value); C = % genome represented by the first ‘n’ scaffolds, following a karyotype of 2n = 36 for this species, estimated as a mode from the ancestral species number of chromosomes (Genome on a Tree—GoaT; tax_name(*Plestiodon gilberti*); Challis et al. 2023).

### Mitochondrial genome assembly

We assembled the mitochondrial genome of *P. gilberti* from the PacBio HiFi reads using the reference-guided pipeline MitoHiFi (Uliano-Silva et al. 2023). The mitochondrial genome of a *Plestiodon capito* (NCBI:MK370740.1) was used as the reference sequence. Because the run did not converge, we extracted the reads that aligned to the *P. capito* reference and conducted a de novo assembly using HiFiasm with default parameters, keeping the primary assembly output (bp.p_ctg). This resulted in a single circular contig. We then annotated the final mitochondrial sequence using MitoFinder (Allio et al. 2020). After completion of the nuclear genome, we searched for matches of the resulting mitochondrial assembly sequence in the nuclear genome assembly using BLAST+ (Camacho et al. 2009) and filtered out contigs and scaffolds from the nuclear genome with a percentage of sequence identity > 99% and size smaller than the mitochondrial assembly sequence.

### Synteny assessment and identification of the X chromosome

Skinks generally have XY sex determination (Gamble et al. 2015), although there are examples of sex reversal (Dissanayake et al. 2021) and environmental sex determination under extreme conditions (Hill et al. 2018). We assessed the synteny of the *P. gilberti* genome with the genome of the distantly related Australian species, the eastern three-lined skink *Acritoscincus duperreyi*, which has well-identified sex chromosomes (Dissanayake et al. 2020), to identify the putative X chromosome for *P. gilberti*. As the sequenced *P. gilberti* was a female, we could not directly identify the sex chromosomes based on differences in sequence coverage. To assess synteny, we aligned the *A. duperreyi* genome (BASDU_1.0) to the *P. gilberti* haplotype 2 assembly using minimap2 (Li 2018) with the asm20 preset. We used the package Asynt (available at https://github.com/simonhmartin/asynt) to plot the results after removing low identity (<5%), low quality (< 60), and short alignments (< 10 kb).

### Genome annotation

We annotated the reference genome using the NCBI Eukaryotic Genome Annotation Pipeline v0.3.2-alpha (hereafter, ‘EGAPX’) which is published in the NCBI RefSeq database (O'leary et al. 2015) and accessible through the NCBI github page (‘ncbi/egapx’). We characterized annotation features by aligning transcripts and proteins from related taxa in the RefSeq database using BLAST (Camacho et al. 2009), and identified novel, species-specific RNAseq reads from this assembly using the alignment software STAR (Dobin et al. 2012). Additional features were predicted using Hidden Markov Model-based gene models using the NCBI Gnomon software (Thibaud-Nissen et al. 2016). We evaluated the quality and completeness of our annotation by comparing the generated list of EGAPX proteins to eukaryotes (odb12), tetrapods (odb10), and squamates (odb12) using BUSCO (Manni et al. 2021). We report the number of annotation features and the BUSCO results in Table 2.

**Table 2:** Sequencing and assembly statistics, and accession numbers for the *Plestiodon gilberti* reference genome.

Bio Projects & Vouchers	CCGP NCBI BioProject		PRJNA720569				
	Genera NCBI BioProject			PRJNA765824			
	Species NCBI BioProject			PRJNA873785			
	NCBI BioSample			SAMN30523592			
	Transcriptome NCBI BioProject			PRJNA1238370			
	NCBI Transcriptome BioSamples			SAMN47472546; SAMN47472545; SAMN47472544; SAMN47472543; SAMN47472542			
	Specimen identification			HBS 135693			
	NCBI Genome accessions			**Haplotype 1**		**Haplotype 2**	
	Assembly accession			JANXHV000000000		JANXHW000000000	
	Genome sequences			GCA_026170395.1		GCA_026170595.1	
Genome Sequence	PacBio HiFi reads	**Run**	1 PACBIO_SMRT (Sequel II) run: 4.7 M spots, 86.8G bases, 66.6Gb				
		**Accession**		SRX19143337			
	Omni-C Illumina reads	**Run**		2 ILLUMINA (Illumina NovaSeq 6000) runs: 189.9 M spots, 87.5G bases, 28.9Gb			
		**Accession**		SRX19143338, SRX19143339			
Transcriptome Sequence	**Tissue type**			**SRA**		**Accession No.**	
	Muscle			SRS24957535		SAMN47472546	
	Lung			SRS24957534		SAMN47472545	
	Liver			SRS24957533		SAMN47472544	
	Heart			SRS24957532		SAMN47472543	
	Brain			SRS24957531		SAMN47472541	
Genome Assembly Quality Metrics	Assembly identifier (Quality code^*^)				rPleGil1(8.8.P8.Q60.C)		
	HiFi Read coverage				56.06X		
				**Haplotype 1**		**Haplotype 2**	
	Number of contigs			62		54	
	Contig N50 (bp)			127 845 512		127 798 174	
	Contig NG50			127 845 512		127 798 174	
	Longest contigs			217 121 865		213 491 989	
	Number of scaffolds			50		39	
	Scaffold N50			184 425 444		231 322 181	
	Scaffold NG50 §			184 425 444		231 322 181	
	Largest scaffold			267 325 462		310 869 391	
	Size of final assembly			1 447 229 999		1 571 222 493	
	Phased block NG50 §			127 845 512		127 798 174	
	Gaps per Gbp (# Gaps)			8(12)		10(15)	
	Indel QV (Frame shift)			49.50898958		49.18994365	
	Base pair QV			60.1737		60.1938	
					Full assembly = 60.1842		
	k-mer completeness			89.4117		97.073	
					Full assembly = 99.2587		
	BUSCO completeness^*^^*^		**C**	**S**	**D**	**F**	**M**
	(tetrapoda_odb10) *n* = 5310	P‡	89.30%	88.50%	0.80%	1.10%	9.60%
		A‡	97.20%	96.60%	0.60%	1.00%	1.80%
	Organelles			1 Partial/complete mitochondrial sequence		JANXHV010000050.1	
Genome Annotation Quality Metrics				**Count of features**			
	Genes			18,697			
	Transcripts			34,009			
	mRNA			32,582			
	IncRNA			1,404			
	CDSs			32,586			
	BUSCO completeness		**C**	**S**	**D**	**F**	**M**
	(eukaryota_odb12) *n* = 129		93.80%	72.90%	20.90%	3.10%	3.10%
	(tetrapoda_odb10) *n* = 5,310		91.50%	55.70%	35.80%	0.30%	8.20%
	(squamata_odb12) *n* = 11,294		91.50%	56.40%	35.10%	0.30%	8.20%

^§^Read coverage and NGx statistics have been calculated based on the estimated genome size of 1.7 Gb.

‡ (H1) Haplotype 1 and (H2) haplotype 2 assembly values.

^*^Assembly quality code x.y.P.Q.C derived notation, from (Rhie et al. 2021). x = log10[contig NG50]; y = log10[scaffold NG50]; P = log10 [phased block NG50]; Q = Phred base accuracy QV (Quality value); C = % genome represented by the first ‘n’ scaffolds, following a known karyotype for this species of 2n = 26 (Genome on a Tree—query(plestiodon gilberti); Challis et al. 2023). Quality code for all the assembly denoted by primary assembly (GCA_026170395.1)

^*^^*^BUSCO Scores. Complete BUSCOs (C). Complete and single-copy BUSCOs (S). Complete and duplicated BUSCOs (D). Fragmented BUSCOs (F). Missing BUSCOs (M).

## Results

### Sequencing data

**Figure 2:**
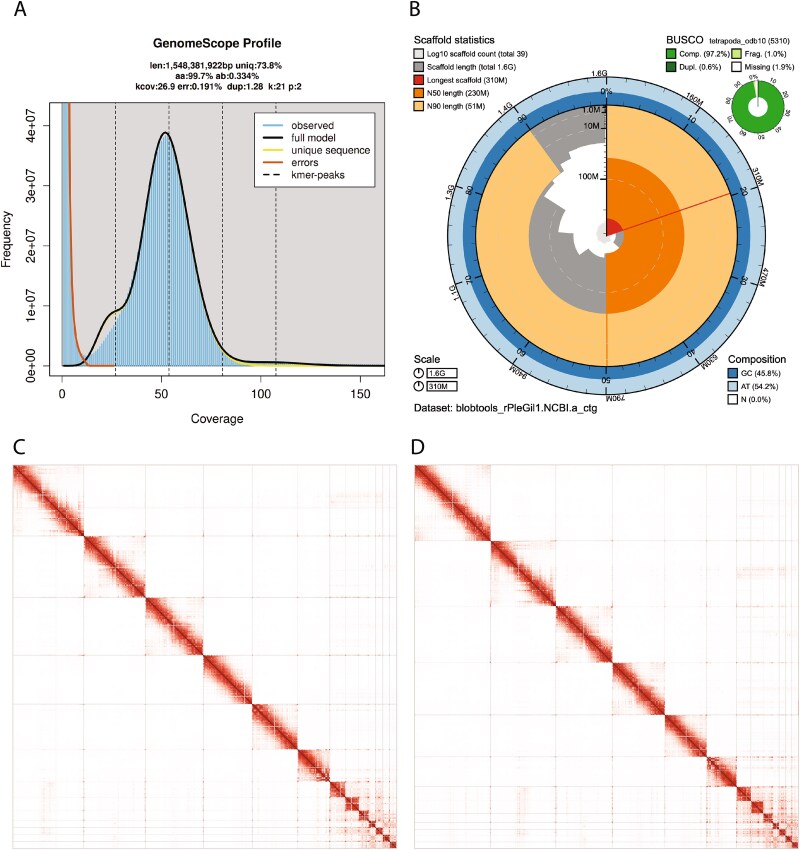
Visual overview of genome assembly metrics. A) K-mer spectra output generated from PacBio HiFi data without adapters using GenomeScope2.0. The bimodal pattern observed corresponds to a diploid genome. K-mers covered at lower coverage and low frequency correspond to differences between haplotypes, whereas the higher coverage and high frequency k-mers correspond to the similarities between haplotypes. B) BlobToolkit snail plot showing a graphical representation of the quality metrics presented in Table 2 for the *Plestiodon gilberti* primary assembly (haplotype 1; GenBank accession no. GCA_026170395.1). The plot circle represents the full size of the assembly. From the inside-out, the central plot covers length-related metrics. The red line represents the size of the longest scaffold; all other scaffolds are arranged in size-order moving clockwise around the plot and drawn in gray starting from the outside of the central plot. Dark and light orange arcs show the scaffold N50 and scaffold N90 values. The central light gray spiral shows the cumulative scaffold count with a white line at each order of magnitude. White regions in this area reflect the proportion of ns in the assembly. The dark vs. light blue area around it shows mean, maximum and minimum GS vs. AT content at 0.1% intervals (Challis et al. 2020). For BUSCO summary data, the definitions are ‘complete and single copy’ (comp.) or ‘complete and duplicated’ (Dupl.). (C-D) Omni-C contact maps for the haplotype 1 (2C) and haplotype 2 (2D) assemblies generated with PretextSnapshot. Omni-C contact maps translate proximity of genomic regions in 3-D space to contiguous linear organization. Each line in the contact map corresponds to sequencing data supporting the linkage (or join) between two of such regions. Scaffolds are separated by black lines and higher density corresponds to high levels of fragmentation.

The Omni-C library generated 289.96 million read pairs and the PacBio HiFi library generated 4.65 million reads. The PacBio HiFi sequences yielded ~56X genome coverage and had an N50 read length of 18 307 bp; a minimum read length of 1 bp; a mean read length of 18,118 bp; and a maximum read length of 65,001 bp (refer to Supplementary Fig. S1 for read length distribution). Based on the PacBio HiFi data, Genomescope 2.0 estimated a genome size of 1.55 Gb, a 0.191% sequencing error rate, and 0.334% heterozygosity. The k-mer spectrum shows a unimodal distribution with a major coverage peak at ~ 50-fold coverage (Fig. 2A). Sequencing of RNA libraries for brain, heart, liver, lung, and muscle generated 50.8 M, 50.0 M, 48.2 M, 56.2 M, and 58.5 M read pairs, respectively.

### Nuclear genome assembly

The final assembly (rPleGil1.0) consists of two phased haplotypes that differ in size by ~ 124 Mb (Table 2)—haplotype 2 is the larger of the two assemblies (~1.57 Gb) and more closely matches the estimated size from GenomeScope 2.0 (Fig. 2A). A list of assembly statistics and quality metrics for both haplotypes is reported in Table 2. Figure 2B provides a graphical representation of the quality metrics for haplotype 2, which has the higher BUSCO completeness score (97.2%) of the two assemblies (Supplementary Fig. S2 displays the same graph for haplotype 1).

During manual curation we made a total of 12 joins (6 per haplotype) and 2 breaks (1 per haplotype) based on the Omni-C contact map signal. We filtered out 1 contig corresponding to contamination from *Babesia bigemina* (Phylum: Apicomplexa; size 44 158 bp) and 2 contigs corresponding to mitochondrial contamination. No further contigs were removed or modified. Omni-C contact maps with chromosome-length scaffolds are shown in Fig. 2C and D, with both indicating highly contiguous assemblies. We have deposited the genome assembly on NCBI GenBank (refer to Table 2 and Data Availability for details).

### Mitochondrial assembly

We assembled a mitochondrial genome for *P. gilberti* with MitoHiFi and annotated the final sequence using MitoFinder. The sequence is comprised of 17,466 bp, with a base composition of A = 24.44%, C = 14.82%, G = 29.29%, and T = 31.46%. It contains two ribosomal RNAs, 22 unique transfer RNAs, and 13 protein coding genes.

### Synteny assessment and identification of the putative X chromosome

**Figure 3:**
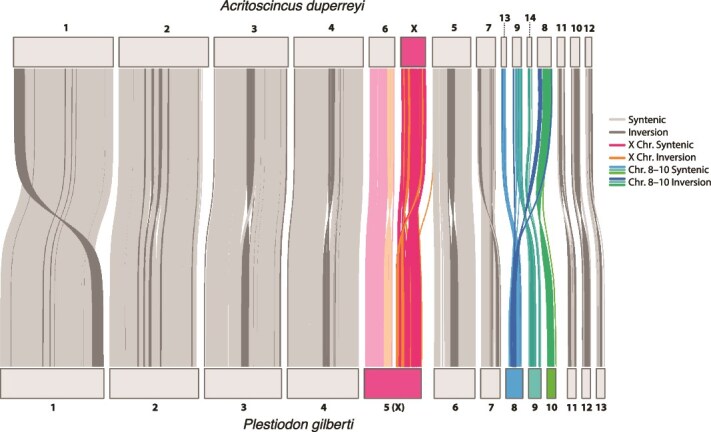
Comparison of the synteny between the new genome assembly of *Plestiodon gilberti* (based on haplotype 2, GenBank assembly accession no. GCA_026170595.1) and the reference genome of the Australian eastern three-lined skink *Acritoscincus duperreyi* (GenBank assembly accession no. GCA_041722995.2; Dissanayake et al. 2020). The numbered horizontal bars represent the chromosomes, with connectors displaying syntenic and inverted alignments between the two species. The first half of *P. gilberti* chromosome 5 aligned to *A. duperreyi* chromosome 6 (light pink), an autosome, whereas the second half aligned to the *A. duperreyi* X chromosome (dark pink).

The chromosomes of *P. gilberti* are highly syntenic with those of *A. duperreyi* (Fig. 3), with most of the autosomes containing a large, inverted region near the center. Smaller chromosomes had a higher degree of rearrangement relative to the six largest chromosomes. For example, our analysis indicates that *P. gilberti* chromosome 9 and *A. duperreyi* chromosomes 8, 9, and 13 are re-arrangements of the same ancestral chromosomes. The *A. duperreyi* X chromosome corresponds to the second half of the larger *P. gilberti* chromosome 5 and displayed substantial rearrangements and inversions. The other half of the *P. gilberti* chromosome 5 aligned to *A. duperreyi* chromosome 6, an autosome. It is not clear if this represents a fusion or division of the ancestral sex chromosome, although two other skink species (*Tiliqua scincoides,* an Australian species and *Cryptoblepharus egeriae* a Christmas Island endemic) are more similar to *A. duperreyi* than *P. gilbert*, suggesting that the *P. gilberti* fusion is derived (results not shown).

### Genome annotation

Our final genome annotation included 18,697 genes, with a tetrapod BUSCO completeness of 91.5%. A list of annotation statistics and BUSCO score breakdowns for eukaryotes, tetrapods, and squamates is reported in Table 2. Additional BUSCO assessment results for RNAseq reads are provided in Supplementary Fig. S3.

## Discussion

Currently there are 106 species of lizard, including this new genome for *P. gilberti*, that have either chromosome- (*n* = 37), scaffold-, (*n* = 56), or contig-level (*n* = 13) assemblies (https://www.ncbi.nlm.nih.gov/datasets/genome; accessed on 4 December 2024), including 10 species drawn from 7 genera of scincid lizards from Australia (genera *Acritoscincus*, *Cryptoblepharus*, *Lerista*, and *Tiliqua)*, sub-Saharan Africa (genera *Mochlus* and *Trachylepis*), the Caribbean region (genus *Spondylurus*), and now North America (this genome for *Plestiodon*)*.* Of these 10 skink species with sequenced genomes, sizes range from ~ 1.21 to 1.74 Gb, with the new *P. gilberti* ~ 1.57 Gb genome being close to the average size of ~ 1.52 Gb. The most recent common ancestor of these sequenced lineages dates to ~ 67 Ma (https://timetree.org, accessed on 02 February 2025: Kumar et al. 2022), indicating that the available genomes represent skinks spanning the most deeply divergent lineages within the Scincidae from disparate parts of the world. Even with the limited number of skink genomes available, there is evidence of substantial chromosomal rearrangements and inversions, including on the X chromosome, and that rearrangements have occurred more frequently on the smaller vs. the larger chromosomes (Fig. 3).

This new reference genome for *P. gilberti*, combined with the CCGP’s resequencing data for *P. gilberti* and its sister species the Western Skink *P. skiltonianus*, will also qualitatively increase our understanding of genetic structure and diversification across California while providing opportunities to identify the genes and genetic mechanisms underlying body size evolution and the ontogeny of color pattern (Fig. 1). Large size and uniform adult coloration are derived states for the *P. skiltonianus* complex (Richmond and Reeder 2002), with size-assortative mating being a primary determinant of reproductive isolation between populations occupying different thermal niches (Richmond and Jockusch 2007; Richmond et al. 2011; Wogan and Richmond 2015). Understanding the genetic underpinnings of these traits and their association to thermal biology stands to broaden our understanding of the links between natural and sexual selection and speciation, particularly in ectotherms like squamate reptiles. The CCGP resequencing data will also enable in-depth studies of the lasting effects of ancient hybridization, including whether introgression is restricted to localized parts of the genome, whether introgressed variants are maintained within a recipient species because of selection, and why gene movement has been largely unidirectional between species.

The more general goals of the data being generated for the *P. skiltonianus* group in the CCGP relate to questions addressing the adaptive capacity of plants and animals to track changing climate. For example, does sufficient standing genetic variation exist to allow skink populations to adapt to changing conditions if species are unable to shift their ranges (Jones et al. 2012; Miller et al. 2019; Todesco et al. 2020; Roberts Kingman et al. 2021), or can interbreeding between ecologically-differentiated populations of closely related species introduce new genetic variants that potentially increase fitness under a warming climate? If ‘adaptive introgression’ is supported, it has the potential to allow populations and species to avoid extirpation/extinction under a rapidly warming climate by allowing them to expand their geographic range or occupy novel niches, a process that may be critical to preserving biodiversity over the long term. Given their history of hybridization, and equipped with this new genome resource, California’s *Plestiodon* skinks are now poised to help us determine whether gene flow can provide critical adaptive variation not only within a single species, but also between close relatives.

## Supplementary Material

JOH-2025-044_R1_suppfile_esaf040

## Data Availability

Data generated for the assembly in this study are available under NCBI BioProject PRJNA720569. NCBI BioSample, Accession, and Short Read Archive (SAR) identifiers are presented in Table 2. Assembly scripts and other data for the analyses presented can be found at the following GitHub repository: www.github.com/ccgproject/ccgp_assembly.
