# Supplementary material for: A chromosome-level reference genome assembly for Gilbert’s skink Plestiodon gilberti
Source: J Hered. 2025 Jun 23;117(1):115–25. doi: 10.1093/jhered/esaf040 (PMC12767197; doi:10.1093/jhered/esaf040)
Supplement: JOH-2025-044_R1_suppfile_esaf040 [file joh-2025-044_r1_suppfile_esaf040.docx]

**Supplementary Figures**

**Title**: A chromosome-level reference genome assembly for Gilbert’s skink *Plestiodon gilberti*

**Authors**: Jonathan Q. Richmond^1*^, Merly Escalona^2^, Mohan P. A. Marimuthu^3^, Oanh Nguyen^3^, Samuel Sacco^4^, Eric Beraut^4^, Erin Toffelmier^5,6^, Benjamin R. Karin^7^, Robert D. Cooper^5,6^, Robert N. Fisher^1^, Ian J. Wang^7,8^, H. Bradley Shaffer^5,6^

**Author affiliations:**

^1^U.S. Geological Survey, 4165 Spruance Rd. Suite 200, San Diego, CA 92101 USA; ^2^Department of Biomolecular Engineering, University of California, Santa Cruz, CA 95064, USA; ^3^DNA Technologies and Expression Analysis Core Laboratory, Genome Center, University of California, Davis, CA 95616, USA; ^4^Department of Ecology and Evolutionary Biology, University of California, Santa Cruz, CA 95064, USA; ^5^Department of Ecology & Evolutionary Biology, University of California, Los Angeles, CA 90095-7239 USA; ^6^La Kretz Center for California Conservation Science, Institute of the Environment and Sustainability, University of California, Los Angeles, CA 90095-7239 USA; ^7^Department of Environmental Science, Policy, and Management, University of California, Berkeley, CA 94720, USA; ^8^Museum of Vertebrate Zoology, University of California, Berkeley, CA 94720, USA

Address correspondence to J.Q. Richmond at the address above, or email***:*** [jrichmond@usgs.gov](mailto:jrichmond@usgs.gov)

Any use of trade, product, or firm names is for descriptive purposes only and does not imply endorsement by the U.S. government.

**Figure S1**. The distribution of PacBio HiFi sequencing read lengths for *Plestiodon gilberti*. The sequences yielded ~56X genome coverage and had a mean read length of 18,118 base pairs.


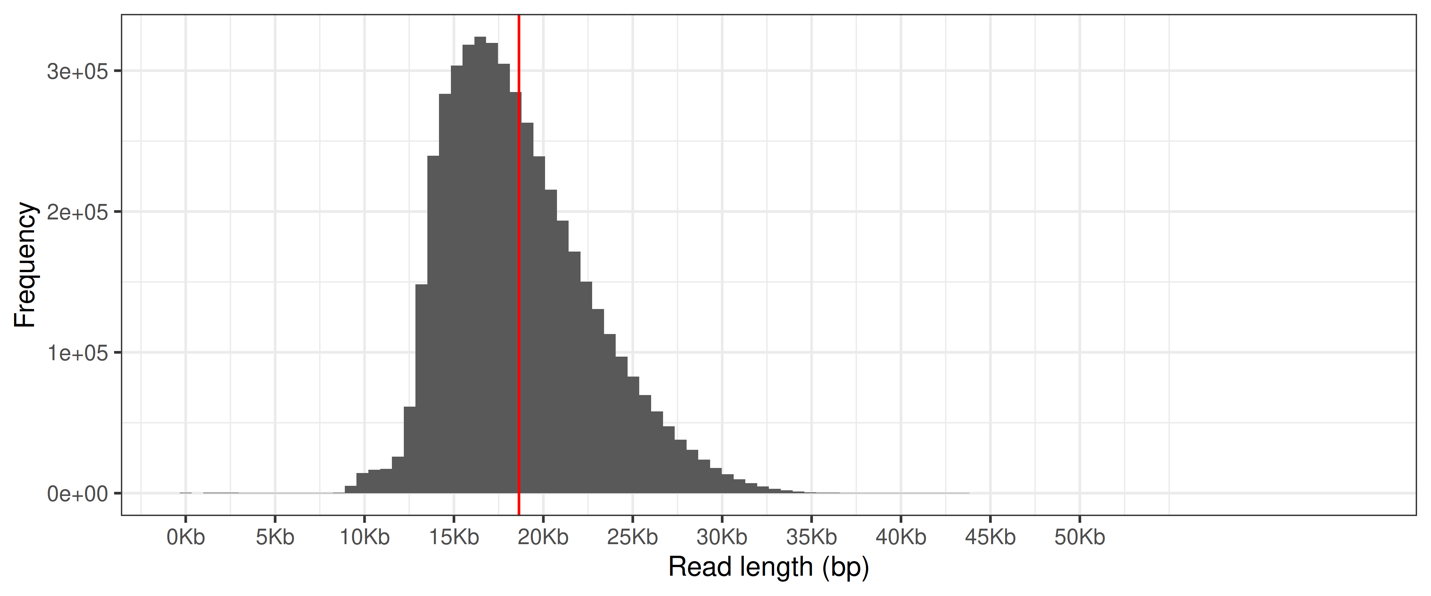


**Figure S2**. B) BlobToolkit Snail plot representing the quality metrics presented in Table 2 for the *Plestiodon gilberti* haplotype 1 assembly (GenBank accession no. GCA_026170395.1). The plot circle represents the full size of the assembly. From the inside-out, the central plot covers length-related metrics. The red line represents the size of the longest scaffold; all other scaffolds are arranged in size-order moving clockwise around the plot and drawn in grey starting from the outside of the central plot. Dark and light orange arcs show the scaffold N50 and scaffold N90 values. The central light grey spiral shows the cumulative scaffold count with a white line at each order of magnitude. White regions in this area reflect the proportion of Ns in the assembly. The dark vs. light blue area around it shows mean, maximum and minimum GS vs. AT content at 0.1% intervals (Challis et al. 2020). For the Busco summary data, definitions are ‘complete and single copy’ (Comp.) and ‘complete and duplicated (Dupl.)


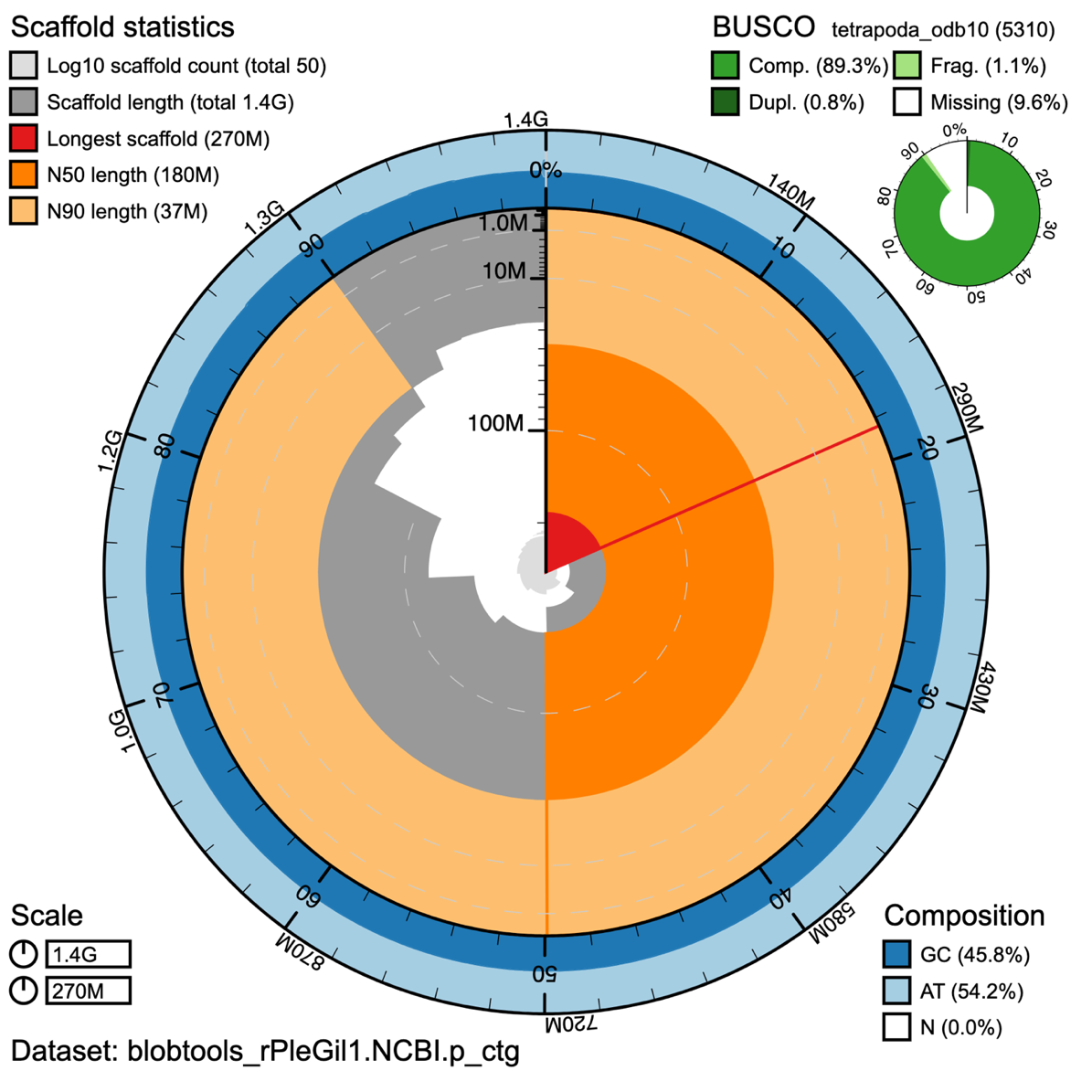


**Figure S3**. Additional BUSCO assessment results based on RNAseq reads for *Plestiodon gilberti*.


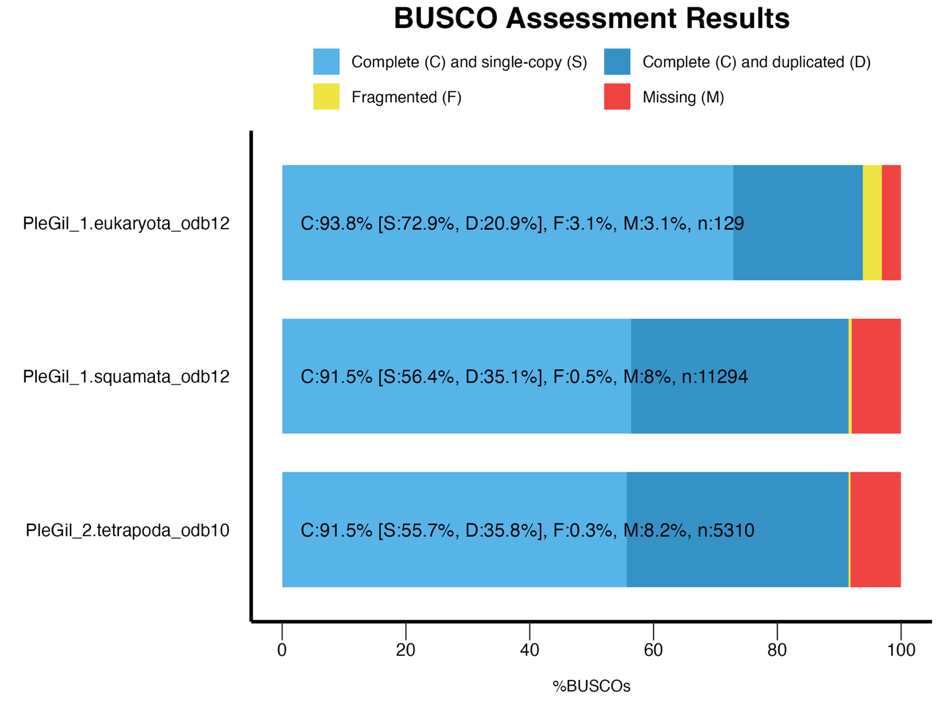


# **References**

Challis R, Richards E, Rajan J, Cochrane G, Blaxter M. 2020 BlobToolKit – Interactive quality assessment of genome assemblies. G3 Genes|Genomes|Genetics. 10:1361-1374.
